# Effects of lactate administration on mitochondrial enzyme activity and monocarboxylate transporters in mouse skeletal muscle

**DOI:** 10.14814/phy2.14224

**Published:** 2019-09-11

**Authors:** Kenya Takahashi, Yu Kitaoka, Yutaka Matsunaga, Hideo Hatta

**Affiliations:** ^1^ Department of Sports Sciences The University of Tokyo Tokyo Japan; ^2^ Department of Human Sciences Kanagawa University Kanagawa Japan

**Keywords:** Lactate, mitochondria, monocarboxylate transporter, skeletal muscle

## Abstract

Growing evidence shows that lactate is not merely an intermediate metabolite, but also a potential signaling molecule. However, whether daily lactate administration induces physiological adaptations in skeletal muscle remains to be elucidated. In this study, we first investigated the effects of daily lactate administration (equivalent to 1 g/kg of body weight) for 3 weeks on mitochondrial adaptations in skeletal muscle. We demonstrated that 3‐week lactate administration increased mitochondrial enzyme activity (citrate synthase, 3‐hydroxyacyl CoA dehydrogenase, and cytochrome c oxidase) in the plantaris muscle, but not in the soleus muscle. MCT1 and MCT4 protein contents were not different after 3‐week lactate administration. Next, we examined whether lactate administration enhances training‐induced adaptations in skeletal muscle. Lactate administration prior to endurance exercise training (treadmill running, 20 m/min, 60 min/day), which increased blood lactate concentration during exercise, enhanced training‐induced mitochondrial enzyme activity in the skeletal muscle after 3 weeks. MCT protein content and blood lactate removal were not different after 3‐week lactate administration with exercise training compared to exercise training alone. In a single bout experiment, lactate administration did not change the phosphorylation state of AMPK, ACC, p38 MAPK, and CaMKII in skeletal muscle. Our results suggest that lactate can be a key factor for exercise‐induced mitochondrial adaptations, and that the efficacy of high‐intensity training is, at least partly, attributed to elevated blood lactate concentration.

## Introduction

Mitochondria oxidize substrates for producing the cell fuel ATP. Given the central role of mitochondria for exercise performance (Fitts et al. 1975) and health (Nasrallah and Horvath 2014), promoting muscle mitochondrial adaptation is important not only for athletes but also for general public. Although it is well established that exercise training induces mitochondrial adaptation in skeletal muscle (Holloszy 1967), the mechanisms which mediate mitochondrial adaptation are not fully unveiled. In recent years, high‐intensity exercise training, accompanied by an elevation of blood lactate concentration, has attracted considerable attention as a time‐efficient alternative to conventional endurance exercise training for inducing mitochondrial adaptations (Gibala et al. 2012; MacInnis and Gibala 2017).

Lactate had been considered a waste product of glycolysis, but is now recognized as an oxidizable substrate, as well as a potential signaling molecule that induces beneficial adaptations in various tissues (Brooks 2018; Ferguson et al. 2018). We previously reported that high‐intensity exercise training increased mitochondrial adaptations (Hoshino et al. 2013), and that decreased lactate accumulation during high‐intensity exercise after administrating dichloroacetate (DCA), an activator of pyruvate dehydrogenase, attenuated training‐induced mitochondrial adaptations in mouse skeletal muscle (Hoshino et al. 2015), suggesting that lactate plays an important role in exercise‐induced mitochondrial adaptations. Hashimoto et al. demonstrated that incubation of L6 cells with lactate increased mRNA levels of peroxisome proliferator‐activated receptor gamma coactivator 1‐alpha (PGC‐1*α*), which is a master regulator of mitochondrial adaptations, and mitochondrial protein content (Hashimoto et al. 2007). We reported that the expression of PGC‐1*α* mRNA was increased after lactate injection in mouse skeletal muscle (Kitaoka et al. 2016). Mitochondrial adaptations appear to result from the cumulative effects of repetitive increases in mRNAs (Perry et al. 2010). Therefore, we hypothesized that daily lactate administration, which increases circulating lactate, induces mitochondrial adaptations in skeletal muscle.

Lactate transport across the plasma membrane occurs via monocarboxylate transporters (MCT) (Bonen, 2000; Kitaoka et al. 2012). Among a family of MCTs (MCT1‐14), MCT1 and MCT4 are thought to play an important role in skeletal muscle (Bonen, 2000). MCT1 is predominantly presented in the oxidative muscles and mainly facilitates lactate uptake, whereas MCT4 is abundant in the glycolytic muscles and mainly mediates lactate efflux from skeletal muscle (Dimmer et al. 2000; Bonen, 2001). Our group reported that MCT protein content in equine skeletal muscle was associated with exercise performance (Kitaoka et al. 2009; Kitaoka et al. 2011), suggesting an important role of MCT for improving exercise performance. Previous studies have showed that high‐intensity exercise training is required for increasing both MCT1 and MCT4 protein contents in skeletal muscle (Pilegaard et al. 1999; Dubouchaud et al. 2000; Thomas et al. 2012). These findings led us to the hypothesis that elevated lactate concentration by daily lactate administration increases MCT protein content in skeletal muscle. Increases in MCT protein content and mitochondrial oxidative capacity by exercise training lead to greater transport and oxidation of lactate (Donovan and Brooks 1983; Bonen 2001). Therefore, to assess whether lactate administration prior to exercise training increased lactate removal from circulation, we performed lactate tolerance test, measuring blood lactate level during sedentary period after lactate injection.

In this study, we first assessed the effects of daily lactate administration for 21 consecutive days on mitochondrial enzyme activity and lactate transporter proteins. Next, we examined whether lactate administration additively enhances exercise‐induced adaptations in skeletal muscle. We also performed lactate tolerance test after the training period. Furthermore, we investigated the effects of a single lactate administration on the responses of intracellular signaling cascades associated with mitochondrial adaptations. We assessed oxidative phenotype muscle, a principal site of lactate oxidation, and glycolytic phenotype muscle, a principal site of lactate production, because their differences in lactate metabolism were expected to cause different adaptations.

## Methods

### Animals

Male ICR mice (8–10 weeks old; CLEA Japan, Tokyo) were used throughout this study. Mice were kept on a 12:12‐h light–dark cycle (dark: 7:00 am to 7:00 pm) in an air‐conditioned room (22°C). All mice were provided with standard chow (MF, Oriental Yeast, Tokyo, Japan) and water ad libitum during the experimental periods. All experiments were approved by the Animal Experimental Committee of The University of Tokyo (no. 27‐14).

### Experimental design

#### Experiment 1: effects of daily lactate administration

Mice were assigned to a control group (CON; *n* = 6) or a lactate group (LAC; *n* = 6). Animals received either phosphate buffered saline (PBS) or 1 g/kg of body weight of sodium lactate via i.p. injection once a day at 13:00–15:00 for 21 consecutive days. The experimental duration was set according to our previous study (Hoshino et al. 2014). The lactate solution concentration was 200 mg/mL, and the pH was 7.3. On the first day of administration, blood of the LAC group after the administration was collected from the tail vein for the measurement of lactate concentration. The soleus muscle (oxidative phenotype) and plantaris muscle (glycolytic phenotype) were harvested 24 h after the last administration, rapidly frozen in liquid nitrogen, and stored at −80°C until further analysis.

#### Experiment 2: effects of lactate administration prior to endurance exercise training

Mice were allocated to a PBS‐administrated sedentary control group (PBS + Sed; *n* = 9), a PBS‐administered endurance exercise training group (PBS + Tr; *n* = 7), or a lactate‐administered endurance exercise training group (LAC + Tr; *n* = 8). Animals were intraperitoneally administrated PBS or sodium lactate (1 g/kg of body weight) once a day for 21 consecutive days. Mice in the PBS + Tr group and the LAC + Tr group performed treadmill running (speed: 20 m/min, duration: 60 min) immediately after each administration. On the first day of the endurance exercise training, the blood lactate concentration in the PBS + Tr and the LAC + Tr groups was measured to confirm that lactate administration elevated blood lactate concentration during the endurance exercise training. All mice performed a lactate tolerance test (LTT) 24 h after the final administration during sedentary period as described below. At 24 h after LTT, the soleus muscle and plantaris muscle were harvested, rapidly frozen in liquid nitrogen, and stored at −80°C until further analysis.

#### Experiment 3: effects of a single lactate administration with or without endurance exercise on intracellular signaling cascades

Mice were assigned to the following four groups: a PBS‐administrated sedentary group (PBS + SED; *n* = 8), a lactate‐administrated sedentary group (LAC + SED; *n* = 8), a PBS‐administrated exercise group (PBS + EXE; *n* = 8), and a lactate‐administrated exercise group (LAC + EXE; *n* = 8). Immediately after the administration of PBS or sodium lactate (1 g/kg of body weight) via i.p. injection, mice remained sedentary or performed treadmill running at a speed of 20 m/min for 60 min. At 60 min after the administration (i.e., immediately after the endurance exercise), the soleus muscle and plantaris muscle were harvested, rapidly frozen, and stored at −80°C.

### Lactate tolerance test (LTT)

A lactate tolerance test was performed following i.p. injection of sodium lactate (1 g/kg of body weight) during the sedentary period. Blood was taken from the tail before, and 5, 15, 30, 60, and 180 min after administration. The blood lactate concentration was measured using a portable blood lactate analyzer (Lactate Pro 2, Arkray, Kyoto, Japan).

### Mitochondrial enzyme activity

The maximal activities of citrate synthase (CS), *β*‐hydroxyacyl CoA dehydrogenase (*β*‐HAD), and cytochrome c oxidase (COX) were determined in whole muscle homogenates. In brief, whole soleus and plantaris muscles were homogenized in 100 (vol/wt) of 100 mmol/L potassium phosphate buffer. Maximal activities were measured spectrophotometrically, as described previously (Smith 1955; Bass et al. 1969; Srere 1969; Spinazzi et al. 2012).

### Western blotting

Whole muscle samples were homogenized in an ice‐cold RIPA buffer (25 mmol/L Tris‐HCl, pH 7.6, 150 mmol/L NaCl, 1% NP‐40, 1% sodium deoxycholate) supplemented with a protease inhibitor mixture (Complete Mini, ETDA‐free, Roche Applied Science, Indianapolis, IN) and a phosphatase inhibitor mixture (PhosSTOP, Roche Applied Science). Muscle homogenates were rotated on ice for 60 min and centrifuged at 1500*g* at 4°C for 20 min. The total protein content of the samples was determined using the BCA protein assay (Pierce, Rockford, IL). Equal amounts of protein, depending on the protein of interest, were loaded onto sodium dodecyl sulfate‐polyacrylamide gel electrophoresis (SDS‐PAGE) gels (7.5%–12%) and separated by electrophoresis. Proteins were transferred to polyvinylidene difluoride membranes before being blocked for 60 min in 5% bovine serum albumin (BSA) in Tris‐buffered saline with 0.1% Tween‐20 (TBST). Membranes were incubated overnight at 4°C with the following primary antibodies: phosphorylated (p‐)AMPKa [Thr172, no. 2513, Cell Signaling Technology (CST) Japan, Tokyo, Japan], AMPKa (no. 2532, CST Japan), p‐acetyl‐CoA carboxylase (ACC; Ser79, no. 3661, CST Japan), ACC (no. 3662, CST Japan), p‐p38 MAPK (Thr170/Tyr182, no. 9211, CST Japan), p38 MAPK (no. 9212, CST Japan), p‐CaMKII (Thr286, no. 3361, CST Japan), CaMKII (no. 611292, BD Biosciences Japan, Tokyo, Japan). Antibodies against MCT1 (monocarboxylate transporter 1) and MCT4 were raised in rabbits against the C‐terminal region of the respective MCT (Qiagen, Japan), and have been used in previous studies (Yoshida et al. 2004; Enoki et al. 2006; Hoshino et al. 2014). Membranes were then incubated with the appropriate host species‐specific secondary antibody (A106PU or A102PT, American Qualex, San Clemente, Calif., USA) for 60 min before being exposed to a chemiluminescence solution (34080, Thermo Fisher Scientific). Three TBST washes were performed between each step. Blots were scanned and quantified using ChemiDoc XRS (Bio‐Rad Laboratories, Hercules, Calif., USA) and Quantity One (version 4.5.2, Bio‐Rad). Ponceau staining was used to verify consistent loading.

### Statistical analysis

All data are expressed as mean ± standard error of means (SEM). In experiment 1, Student's t‐test was used to examine the effects of lactate administration. In experiment 2, one‐way analysis of variance (ANOVA) was performed, followed by Fisher’s protected least significant difference test. In experiment 3, two‐way ANOVA was performed. For the time course of the blood lactate concentration, two‐way was performed followed by Bonferroni’s multiple comparison test. All statistical analyses were performed by GraphPad Prism (Ver. 7.0, Macintosh, GraphPad Software, La Jolla, CA). Statistically significant differences and trends were defined as *P* values less than 0.05 and less than 0.10, respectively.

## Results

### Effects of daily lactate administration

**Figure 1:**
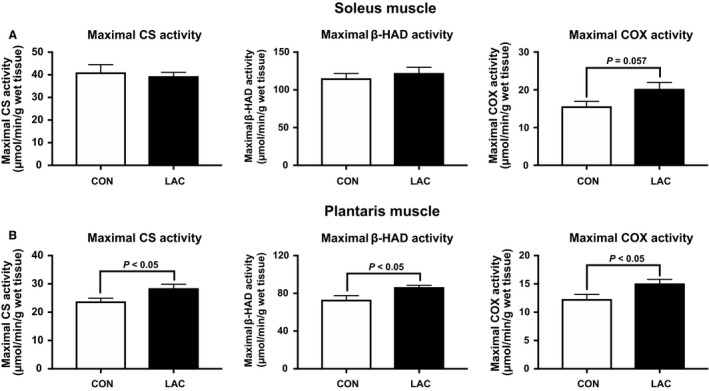
Mitochondrial enzyme activity in Experiment 1. Maximal CS, *β*‐HAD, and COX activities in the soleus muscle (A) and the plantaris muscle (B) after 3 weeks of administration. Data are expressed as mean ± SEM. *n* = 6.

**Figure 2:**
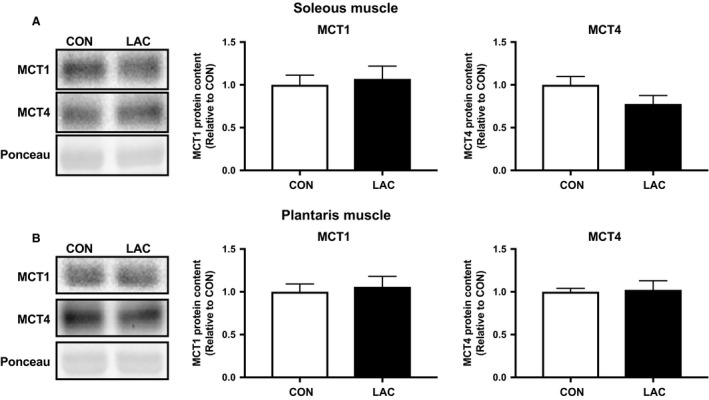
MCT protein content in Experiment 1. MCT1 and MCT4 protein contents in the soleus muscle (A) and the plantaris muscle (B) after 3 weeks of administration. Data are expressed as mean ± SEM. *n* = 6.

We first confirmed the time course changes in blood lactate concentration after lactate injection. Based on our previous report (Kitaoka et al. 2016), we aimed to increase the blood lactate concentration at an upper physiological level (~20 mmol/L). In this study, the peak blood lactate concentration reached 12.7 ± 1.3 mmol/L 15 min after lactate administration. Following the 21 days of the experimental period, body weight and skeletal muscle weight did not differ between the two groups (Table 1). CS, *β*‐HAD, and COX activities in the soleus muscle were not significantly different between groups, although COX activity tended to be higher in the LAC group than in the CON group (*P* = 0.057) (Fig. 1A). In the plantaris muscle, CS, *β*‐HAD, and COX activities were significantly higher in the LAC group than in the CON group (Fig. 1B). The MCT1 and MCT4 protein contents in the soleus muscle (Fig. 2A) and the plantaris muscle (Fig. 2B) were not altered after 3‐week lactate administration.

**Table 1:** Body weight, soleus muscle weight, and plantaris muscle weight following 3 weeks of the sedentary experiment (Experiment 1).

	CON	LAC
Body weight (g)	39.3 ± 0.9	37.3 ± 0.7
Soleus muscle (mg)	16.3 ± 1.5	16.2 ± 1.0
Plantaris muscle (mg)	38.6 ± 1.9	37.4 ± 1.2

Data are expressed as mean ± SEM. *n* = 6.

### Effects of lactate administration prior to endurance exercise training

**Figure 3:**
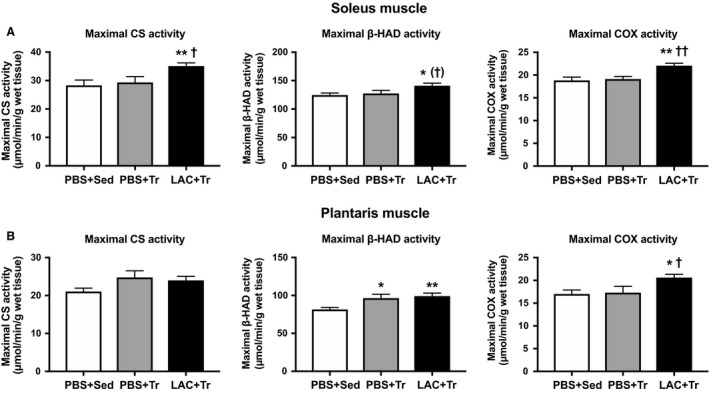
Mitochondrial enzyme activity in Experiment 2. Maximal CS, *β*‐HAD, and COX activities in the soleus muscle (A) and the plantaris muscle (B) after 3 weeks of exercise training experiment. Data are expressed as mean ± SEM. *n* = 7–9 in each group. *P < 0.05, **P < 0.01: significant difference versus PBS + Sed. ^†^ *P* < 0.10, ^†^ *P* < 0.05, ^††^ *P* < 0.01: tend and significant difference versus PBS + Sed.

**Figure 4:**
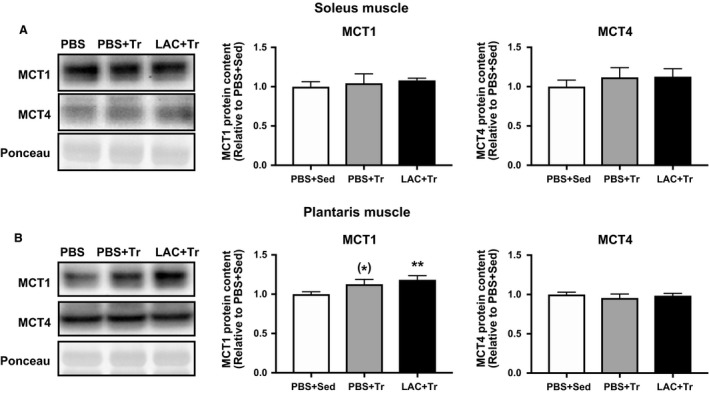
MCT protein content in Experiment 2. MCT1 and MCT4 protein contents in the soleus muscle (A) and the plantaris muscle (B) after 3 weeks of exercise training experiment. Data are expressed as mean ± SEM. *n* = 7–9 in each group. *P < 0.10, **P < 0.01: tend and significant difference versus PBS + Sed.

**Figure 5:**
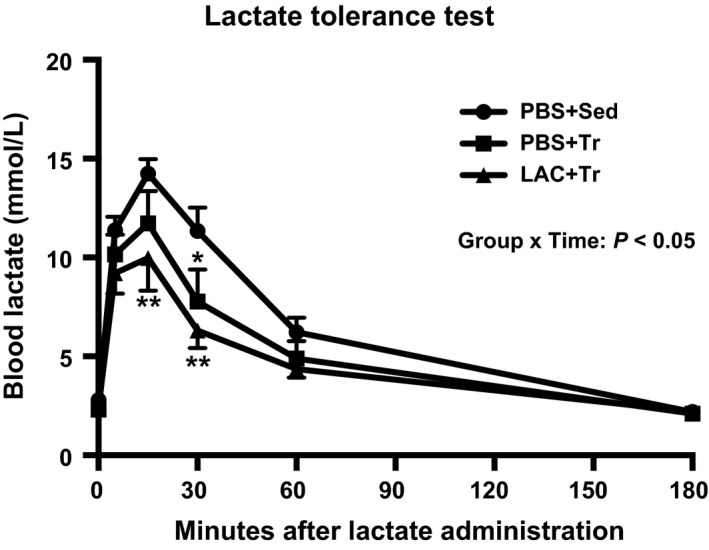
Lactate tolerance test in Experiment 2. Time course of changes in blood lactate concentration during LTT 24 h after the last training session following 3 weeks of the exercise training experiment. Data are expressed as mean ± SEM. *n* = 7–9 in each group. *P < 0.05, **P < 0.01: main effect of endurance exercise training.

The peak blood lactate concentration during endurance exercise was observed 15 min after the administration (i.e., at 15 min of exercise), and was significantly higher in the LAC + Tr group (14.3 ± 2.0 mmol/L) compared with the PBS + Tr group (4.1 ± 0.6 mmol/L). Following the training period, there was no difference in either body weight or skeletal muscle weight among the groups (Table 2). In the soleus muscle, CS and COX activities were significantly higher in the LAC + Tr group than in the PBS + Sed group and the PBS + Tr group. *β*‐HAD activity in the soleus muscle was significantly higher in the LAC + Tr group than in the PBS + Sed group, and there was a positive trend in the LAC + Tr group compared with the PBS + Tr group (Fig. 3A). In the plantaris muscle, CS activity was not significantly different among the groups. However, *β*‐HAD activity was significantly higher in the PBS + Tr and the LAC + Tr groups than in the PBS + Sed group. COX activity in the plantaris muscle was significantly higher in the LAC + Tr group than in the PBS + Sed and the PBS + Tr groups (Fig. 3B). The MCT1 and MCT4 protein contents in the soleus muscle were not significantly different among the groups (Fig. 4A). In the plantaris muscle, the MCT1 protein content was significantly higher in the LAC + Tr group than in the PBS + Sed group, and tended to be higher in the PBS + Tr group than in the PBS + Sed group, although the MCT4 protein content was not significantly different among the groups (Fig. 4B). In the LTT, the PBS + Tr group showed a significantly lower blood lactate concentration at 30 min after lactate administration compared with the PBS + Sed group. Furthermore, the LAC + Tr group showed a significantly lower blood lactate concentration at 15 and 30 min after lactate administration than the PBS + Sed group. However, there was no significant difference between the PBS + Tr group and the LAC + Tr group (Fig. 5).

**Table 2:** Body weight, soleus muscle weight, and plantaris muscle weight following 3 weeks of the exercise training experiment (Experiment 2).

	PBS + Sed	PBS + Tr	LAC + Tr
Body weight (g)	40.1 ± 0.7	38.4 ± 0.5	39.0 ± 0.3
Soleus muscle (mg)	18.2 ± 0.7	18.0 ± 1.3	18.1 ± 1.0
Plantaris muscle (mg)	38.0 ± 1.5	40.5 ± 1.4	36.8 ± 1.7

Data are expressed as mean ± SEM. *n* = 7–9 in each group.

### Effects of a single lactate administration with or without endurance exercise on intracellular signaling cascades

**Figure 6:**
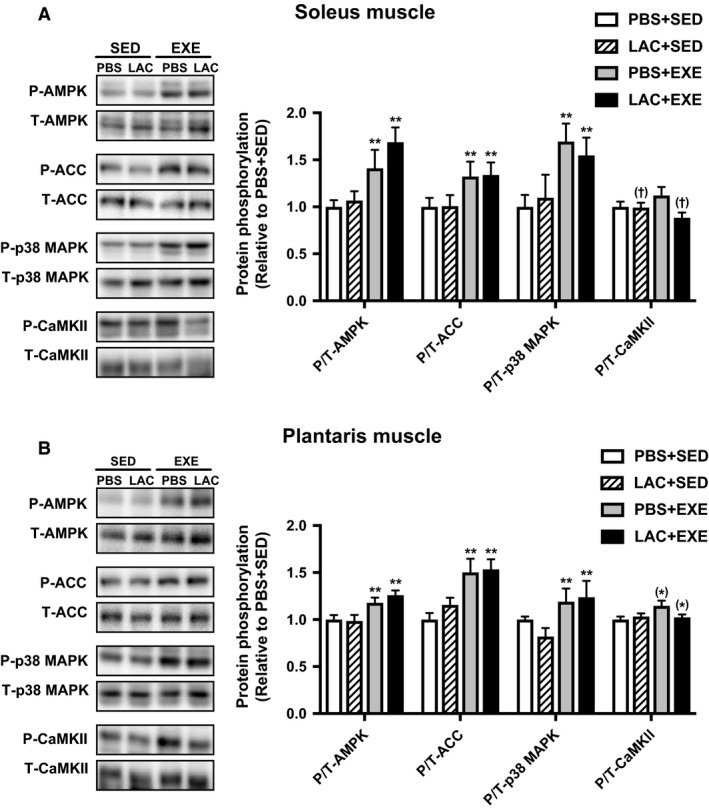
Protein phosphorylation in Experiment 3. Phosphorylation of intramuscular signaling kinase in the soleus muscle (A) and the plantaris muscle (B) 60 min after the administration (i.e., immediately after 60 min of endurance exercise) in the single administration experiment. Data are expressed as mean ± SEM. *n* = 8 in each group. *P < 0.10, *P* < 0.05, **P < 0.01: main effect of endurance exercise. ^(†)^ *P* < 0.10: main effect of lactate administration.

Endurance exercise significantly increased AMPK, ACC, and p38 MAPK phosphorylation in the soleus muscle (Fig. 6A) and the plantaris muscle (Fig. 6B). However, no significant main effects of lactate administration were detected in either the soleus muscle or the plantaris muscle.

## Discussion

### Lactate‐induced mitochondrial adaptation

To the best of our knowledge, this is the first study to show that daily lactate administration induced partial mitochondrial adaptations (i.e., mitochondrial enzyme activity) in skeletal muscle. A previous study reported that increased blood lactate accumulation following ingestion of sodium bicarbonate prior to high‐intensity exercise enhanced PGC‐1*α* mRNA expression in human skeletal muscle (Percival et al. 2015). Another study reported that PGC‐1*α* mRNA expression in human skeletal muscle increased after exercise above the intensity of the lactate threshold (LT), but not below the LT (Tobina et al. 2011). These reports led us to hypothesize that higher blood lactate concentration during exercise leads to greater mitochondrial adaptations in skeletal muscle. In this study, we observed that lactate administration prior to endurance exercise training resulted in higher mitochondrial enzyme activity in the skeletal muscle compared with endurance exercise training with PBS administration. This is in accordance with previous observations that decreased lactate accumulation during high‐intensity exercise attenuated training‐induced mitochondrial adaptations such as enzyme activity and protein content (Hoshino et al. 2015). Altogether, our results suggest that lactate plays an important role for mitochondrial adaptations, and that the efficacy of high‐intensity exercise training may partly be caused by elevated blood lactate concentration.

In this study, lactate‐induced mitochondrial adaptation was varied according to muscle phenotype. In the sedentary experiment, mitochondrial adaptation was greater in the plantaris muscle than in the soleus muscle. A previous study reported that electrical stimulation‐induced mitochondrial adaptation was less in high‐oxidative muscle than in low‐oxidative muscle (Ljubicic and Hood 2009), indicating that greater stimulation is required to induce mitochondrial adaptation in the soleus muscle. Therefore, we speculate that lactate administration alone was not sufficient to induce the adaptation in the soleus muscle. We found that lactate administration prior to endurance exercise training enhanced mitochondrial enzyme activity in the soleus muscle, while endurance exercise training alone had no significant effect on the enzyme activity. Our results suggest that lactate administration enhances exercise‐induced mitochondrial adaptation in the soleus muscle. In the plantaris muscle, the endurance exercise training alone increased mitochondrial enzyme activity, while lactate administration had smaller additive effects, possibly because of lactate production during exercise in glycolytic muscles (Wilson et al. 1998; Dimmer et al. 2000).

### Daily lactate administration on MCT and lactate tolerance test

Lactate is reported to increase MCT1 protein content in L6 cells (Hashimoto et al. 2007). In addition, low‐intensity endurance exercise training increases MCT1 protein content but not MCT4 protein content (Dubouchaud et al. 2000), whereas high‐intensity exercise training increases both MCT1 and MCT4 protein contents in skeletal muscle (Pilegaard et al. 1999; Kitaoka et al. 2011). These observations indicate that an elevated lactate concentration can be a key factor for increasing MCT protein contents. In the present study, however, MCT protein content was not significantly different after 3‐week lactate administration with exercise training compared to exercise training alone. Supporting our current results, a previous study reported no significant differences in MCT1 and MCT4 protein contents following two different training strategies that elicited distinct blood lactate concentrations (Mohr et al. 2007). Moreover, longitudinal data showed no significant correlation between peak blood lactate concentration during exercise training and percentage increase in MCT protein content (Thomas et al. 2012). Collectively, elevated blood lactate concentration does not simply affect MCT protein content in skeletal muscle. However, it should be noted that MCT1 protein content was significantly increased after endurance exercise training with lactate administration, despite no significant increase in MCT1 protein content after endurance exercise training alone, suggesting non‐negligible effect of lactate on MCT1. Further study is required to elucidate the precise mechanisms by which MCT1 expression is regulated.

To assess whether lactate administration prior to exercise training increased lactate removal from circulation, we performed lactate tolerance test. Endurance exercise training resulted in lower blood lactate concentration after lactate injection. Given that lactate transport across the sarcolemmal membrane depends on MCT protein content (Baker et al. 1998) and concentration gradient (Roth and Brooks 1990), the results of the LTT in the present study may be attributed to both mitochondrial enzyme activity and MCT protein content in skeletal muscle. Supporting this view, Thomas et al. reported that a rate of lactate removal from blood after supramaximal exercise was positively correlated with maximal CS activity (Thomas et al. 2004) and the MCT1 protein content in skeletal muscle (Thomas et al. 2005). However, we observed no additive effect of lactate administration in the LTT despite higher mitochondrial enzyme activity. Previous studies reported that rate of lactate turnover was substantially lower during at rest than during exercise (Richter et al. 1988; Van Hall et al. 2003). Since the LTT was performed during sedentary state, we speculate that increased mitochondrial enzyme activity did not alter the rate of lactate removal. It should be noted that lactate circulates through the whole body via the bloodstream (Van Hall 2010), implying that the effects of lactate administration are not limited to skeletal muscle. Previous studies reported that lactate injection increased PGC‐1*α* mRNA in liver (E et al. 2013), and that lactate induced browning of white adipose tissue (Carriere et al. 2014), which increases MCT1 protein content and mitochondria. Thus, the result of the LTT was not solely attributed to the adaptations of skeletal muscle, but also to other organs.

### Effects of a single lactate administration on signaling cascades

To identify the possible mechanisms of the lactate‐induced mitochondrial adaptations, we investigated the phosphorylation state of AMPK, ACC, p38 MAPK, and CaMKII, key kinases for mitochondrial adaptations (Egan and Zierath 2013). Although endurance exercise significantly increased the phosphorylation state of AMPK, ACC, and p38 MAPK in skeletal muscle, lactate administration did not change the phosphorylation state of these kinases. Consistent with our current observations, the phosphorylation state of those kinases in human skeletal muscle was not different after an increase in the blood lactate level during high‐intensity exercise, despite up‐regulation of exercise‐induced PGC‐1*α* mRNA expression (Percival et al. 2015). Furthermore, Hoshino et al. (2015) reported that decreased lactate accumulation during high‐intensity exercise did not significantly change the exercise‐induced phosphorylation state of those kinases. Taken together, those kinases do not appear to be related to lactate‐induced mitochondrial adaptations. At present, mechanisms by which lactate induces mitochondrial adaptations are not thoroughly defined. It has been reported that a selective receptor for lactate, called G‐protein‐coupled receptor 81 (GPR81), which is reported to activate other signaling pathways (Ohno et al. 2018), exists in various tissues, including skeletal muscle (Liu et al. 2009). Therefore, it cannot be ruled out that lactate‐induced mitochondrial adaptations were caused by activation of other pathways. Furthermore, a previous study reported that lactate regulates gene transcription through the inhibition of histone deacetylase (Latham et al. 2012), implying that lactate may activate not only transient signaling pathways but also gene transcription. Moreover, other factors derived from diverse tissues cannot be excluded. For example, a recent study has reported that lactate promotes secretion of TGF‐*β*2, which up‐regulates PGC‐1*α* mRNA expression in skeletal muscle, from adipose tissue (Takahashi et al. 2019). Thus, a future study should clarify whether these earlier findings explain the current observations.

## Conclusions

Daily lactate administration increased mitochondrial enzyme activity in the glycolytic muscle (i.e., plantaris muscle). In addition, lactate administration prior to endurance exercise training enhanced training‐induced mitochondrial enzyme activity in the skeletal muscle. These results suggest that lactate can be a key factor for exercise‐induced mitochondrial adaptations, and that the efficacy of high‐intensity exercise training may be caused in part by higher blood lactate concentration. However, circulating lactate does not seem to increase MCT protein content in skeletal muscle. Furthermore, lactate‐induced mitochondrial adaptations do not appear to be associated with greater activation of the AMPK, p38 MAPK, and CaMKII pathways.

## Conflict of Interest

There is no conflict of interest.
